# Experimental model of VZV reactivation reveals vaccine-induced mechanisms of protection against herpes zoster

**DOI:** 10.64898/2026.09.22.26363709

**Published:** 2026-09-25

**Authors:** Michael J. Johnson, Thao Vu, Emily S. Ford, Lichen Jing, Kimberly Jordan, Mariah Brown, Shaobing Li, Troy Schedin, Megan Crotteau, Jennifer Canniff, Ashlynn Bennett, Neil Weaver, Kerry J. Laing, Seth Frietze, David M. Koelle, Myron J. Levin, Adriana Weinberg

## Abstract

Herpes zoster (HZ), caused by varicella zoster virus (VZV) reactivation, is common in older adults with declining cell-mediated immune protection. A modestly efficacious live attenuated (ZVL) and a highly efficacious recombinant adjuvanted (RZV) zoster vaccine protect against HZ. To uncover the mechanism(s) associated with the superior efficacy of RZV, we developed a controlled human infection model consisting of intradermal administration of attenuated vOka VZV to older adults previously vaccinated with RZV or ZVL. Infiltration of VZV-specific CD4+ and CD8+ T cell clonotypes at the infection site was quicker and higher in RZV than ZVL recipients ≥5 years post-immunization due to the persistence of RZV-induced gE-reactive CD4+ and CD8+ T cell clonotypes in blood. High CD4+ cytotoxic T lymphocyte frequencies in blood on Day 1 post-inoculation predicted control of VZV replication. Persistence of circulating RZV-induced CD4+ and CD8+ CTL and rapid migration to sites of VZV replication are potential mechanisms underlying durable protection against HZ.

## Full Text Availability

The license terms selected by the author(s) for this preprint version do not permit archiving in PMC. The full text is available from the preprint server.

